# Unmasking BRASH Syndrome: A Clinical Challenge in Bradycardia and Renal Dysfunction

**DOI:** 10.7759/cureus.98773

**Published:** 2025-12-08

**Authors:** Ervan Zuhri, Sabrina Erriyanti

**Affiliations:** 1 Cardiology and Vascular Medicine, National Cardiovascular Center Harapan Kita, Jakarta, IDN; 2 Cardiology and Vascular Medicine, PT Pelayaran Nasional Indonesia (PELNI) Hospital, Jakarta, IDN

**Keywords:** bradycardia, brash, hyperkalemia, renal dysfunction, sinus node dysfunction

## Abstract

The term BRASH refers to a clinical constellation characterized by bradycardia, renal dysfunction, atrioventricular (AV) nodal-blocking medication exposure, shock, and hyperkalemia. Although the condition represents a fully reversible cause of symptomatic bradycardia, it frequently goes unrecognized. Prompt identification and a clear understanding of its underlying mechanisms are essential to avoid unnecessary procedures. The syndrome should be suspected when a patient presents with bradycardia in the setting of impaired kidney function and elevated serum potassium, particularly when there is recent or ongoing use of AV nodal-blocking agents. Early recognition is critical because effective management requires addressing each contributing factor simultaneously, rather than treating a single abnormality in isolation.

## Introduction

BRASH syndrome represents a clinical pattern in which marked bradycardia occurs simultaneously with impaired renal function, exposure to atrioventricular (AV) nodal-blocking medications, circulatory instability, and elevated potassium levels. The syndrome develops through the combined effects of hyperkalemia and drugs that slow AV nodal conduction, most commonly beta-blockers or non-dihydropyridine calcium channel blockers. Its presentation is highly variable, ranging from mild reductions in heart rate to profound hemodynamic collapse [1]. Although the condition is fully reversible, it is frequently overlooked in real-world practice, as its individual components are often misattributed to isolated hyperkalemia, medication toxicity, or renal dysfunction rather than recognized as a single unifying syndrome. Recognizing the mechanism behind this syndrome at an early stage is essential to avoid unnecessary procedures, including pacemaker implantation [2].

## Case presentation

A 65-year-old woman with a background of coronary artery disease without significant stenosis, grade 2 diastolic dysfunction with preserved systolic function, hypertension, and stage 5 chronic kidney disease (not yet requiring dialysis) arrived at the emergency department complaining of profound fatigue and atypical chest discomfort. Her chronic medications consisted of clonidine 0.15 mg twice daily, nitroglycerin 2.5 mg twice daily, candesartan 16 mg daily, acetylsalicylic acid 100 mg daily, and simvastatin 40 mg daily. In an attempt to control persistently elevated blood pressure, she began taking diltiazem 100 mg daily on her own two days before presentation.

**Figure 1:**
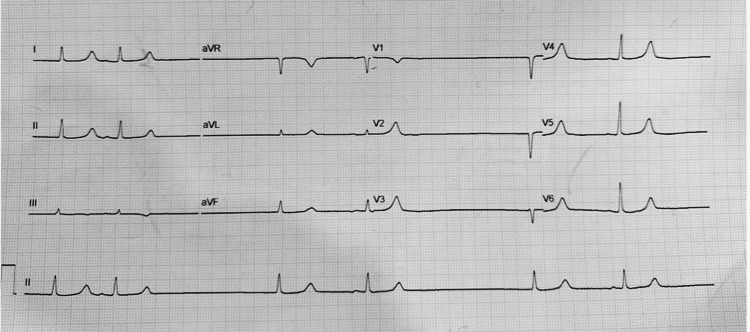
Patient’s ECG at admission showing a sinus pause with junctional escape rhythm.

On arrival, she was conscious but markedly bradycardic, with a heart rate of 36 beats per minute and a blood pressure of 89/52 mmHg. Examination showed generalized weakness, a slow pulse, and cool extremities. Laboratory studies revealed severe hyperkalemia, worsening renal function, metabolic acidosis, and anemia (Table 1). Her initial ECG demonstrated a sinus pause with junctional escape rhythm at 36 beats per minute, without the classic ECG abnormalities typically associated with hyperkalemia (Figure 1). Echocardiography confirmed preserved systolic performance with an ejection fraction of 69% and no regional wall motion abnormalities.

**Table 1:** Patient’s laboratory results. ABGA = arterial blood gas analysis; BUN = blood urea nitrogen; eGFR = estimated glomerular filtration rate

Parameter	Result	Reference range
Potassium	7.7 mEq/L	3.4–4.5 mEq/L
Creatinine	5.8 mg/dL	0.5–1.3 mg/dL
BUN	145 mg/dL	7–30 mg/dL
eGFR	7 mL/minute/1.73m^2^	≥60 mL/minute/1.73m^2^
Hemoglobin	7.1 g/dL	12.0–16.0 g/dL
pH (ABGA)	7.28	7.35–7.45
HCO_3_ (ABGA)	14 mmol/L	23.0–28.0 mmol/L
Base excess (ABGA)	-16.2 mEq/L	-2.5–2.5 mEq/L

**Figure 2:**
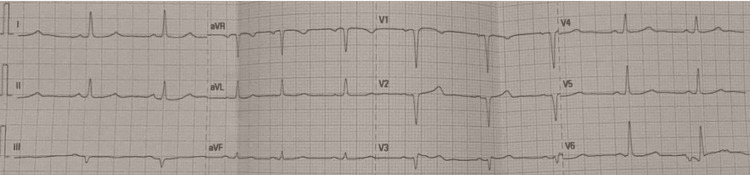
Patient’s ECG after simultaneous treatment showing a normal sinus rhythm.

She received two 1 mg doses of intravenous atropine, which produced only a brief rise in heart rate into the low 40s. Intravenous dopamine infusion and normal saline were started, improving her blood pressure to 98/61 mmHg and increasing her heart rate to 50 beats per minute. In parallel, hyperkalemia was treated with intravenous calcium gluconate (1 g of 10% solution), 10 units of regular insulin with 50 mL of 40% dextrose, followed by urgent hemodialysis. Although exact timestamps were not recorded, clinical improvement occurred shortly after the correction of hyperkalemia. Following hemodialysis, the patient’s serum potassium decreased to 5.8 mmol/L, and a repeat ECG obtained approximately one to two hours later demonstrated restoration of normal sinus rhythm (Figure 2).

Dopamine was weaned off as her hemodynamic status stabilized, and her symptoms progressively improved. She was discharged in stable condition five days later, without any indication for temporary or permanent pacing. A follow-up 24-hour Holter monitor performed three months after discharge showed no evidence of clinically significant bradycardia or sinus node dysfunction.

## Discussion

Sinus node dysfunction occurs when abnormalities within the sinoatrial node disrupt the regularity, rate, or transmission of electrical impulses through the cardiac conduction system. When evaluating a patient with suspected sinus node dysfunction, the initial step is to identify any reversible contributors, such as acute myocardial infarction, thyroid disorders, metabolic or electrolyte disturbances, or medication effects. The use of AV nodal-blocking medications in individuals with hyperkalemia and renal impairment can precipitate BRASH syndrome [1,2].

BRASH syndrome is a fully reversible cause of significant bradycardia. Early recognition of its underlying mechanisms and triggers is essential to ensure appropriate management and avoid unnecessary interventions. In contrast to bradycardia caused solely by hyperkalemia, which typically presents with characteristic ECG changes such as tall T waves, QRS widening, or a sine wave pattern, BRASH may present without these hallmark findings, leading to diagnostic difficulty. This stems from the combined effects of hyperkalemia and additional stressors, including AV nodal-blocking agents or volume depletion in patients with renal dysfunction [3-5]. In such cases, the dominant effect of AV nodal-blocking therapy can mask the classic ECG manifestations of hyperkalemia, as profound bradycardia and slowed conduction obscure expected changes such as peaked T waves or QRS widening.

Pathophysiologically, BRASH develops when AV nodal-blocking medications (e.g., non-dihydropyridine (DHP) calcium channel blockers) intensify the depressant effects of hyperkalemia on cardiac conduction, resulting in marked bradycardia, reduced cardiac output, and diminished renal perfusion. In the setting of advanced renal impairment, hyperkalemia worsens and drug clearance declines, perpetuating a self-reinforcing cycle that further decreases cardiac output and renal blood flow. Without timely intervention, this process can progress to cardiogenic shock or multiorgan failure. Dehydration may accelerate this deterioration by further reducing renal perfusion [3-5].

Our case shares many features typical of BRASH but also includes several distinctive aspects. The patient exhibited severe hyperkalemia (7.7 mEq/L) with sinus pause and a junctional escape rhythm, yet lacked the classic ECG manifestations of hyperkalemia, such as peaked T waves or QRS prolongation. This diagnostic ambiguity is increasingly recognized in BRASH, where the synergistic interplay between AV nodal blockade and hyperkalemia may obscure expected ECG findings. Previous reports highlight a spectrum of severity. Farkas et al. [1] described the prototypical BRASH cycle involving beta-blocker therapy, moderate hyperkalemia, and renal dysfunction, often requiring extended vasopressor support. Gebray et al. [3] documented a case with complete heart block necessitating temporary pacing. Bailuni Neto et al. [4] reported BRASH associated with verapamil requiring repeated dialysis and ongoing hemodynamic instability. Finally, Srivastava et al. [5] demonstrated that even minor electrolyte abnormalities may precipitate shock in predisposed patients.

In contrast, our patient (1) self-initiated diltiazem, a non-DHP calcium channel blocker, only two days before presentation, a rarely reported trigger; (2) had no prior evidence of sinus node dysfunction, with complete recovery of heart rhythm after correcting the underlying metabolic derangements, confirmed by a normal three-month Holter study; and (3) improved without the need for pacemaker implantation, intensive care, or prolonged hospitalization. These findings underscore the importance of early recognition and targeted management in interrupting the BRASH cycle.

Management centers on breaking this cycle by restoring cardiac output and renal perfusion while addressing all contributing factors. This approach includes discontinuing AV nodal-blocking medications, treating hyperkalemia aggressively, and considering dialysis when indicated, not primarily for fluid removal but to correct hyperkalemia and remove accumulated AV nodal-blocking agents [3-5].

## Conclusions

This case highlights several important clinical lessons. First, the absence of classic ECG changes does not rule out significant hyperkalemia or BRASH syndrome. Second, the combination of renal dysfunction and AV nodal-blocking medications should prompt consideration of BRASH, particularly in patients who present with bradycardia and low blood pressure. Third, effective treatment hinges on promptly correcting hyperkalemia, discontinuing AV nodal blockers, and providing appropriate hemodynamic support, often preventing the need for invasive procedures. Overall, BRASH syndrome should be understood as a dynamic and interrelated process rather than an isolated abnormality, requiring rapid, comprehensive management to avoid progression to cardiogenic shock or multiorgan failure.
